# Psychometric properties and factor structure of the Repetitive Thinking Questionnaire: Persian versions of the RTQ-31 and RTQ-10

**DOI:** 10.47626/2237-6089-2020-0058

**Published:** 2021-11-09

**Authors:** Mohsen Hasani, Reza Ahmadi, Omid Saed

**Affiliations:** 1 Zanjan University of Medical Sciences Faculty of Medicine Department of Clinical Psychology Zanjan Iran Department of Clinical Psychology, Faculty of Medicine, Zanjan University of Medical Sciences, Zanjan, Iran.

**Keywords:** Repetitive Thinking Questionnaire, rumination, psychometric properties, factor structure, depression, anxiety

## Abstract

**Introduction:**

Repetitive thinking as a transdiagnostic factor plays an essential role in the development and maintenance of emotional disorders. Two versions of the Repetitive Thinking Questionnaire (RTQ-31 and RTQ-10) are the best-known measures used for assessing repetitive thinking in clinical and non-clinical samples. The present study was conducted to evaluate the psychometric properties and factor structure of Persian versions of them.

**Methods:**

Participants were 592 students assessed with the RTQ-31, the RTQ-10, the Ruminative Response Scale, the Perseverative Thinking Questionnaire, the Beck Depression Inventory-second edition, the Beck Anxiety Inventory, and the Depression, Anxiety, Stress Scale-21. Exploratory and confirmatory factor analysis were used to determine construct validity.

**Results:**

The findings showed that the RTQ-31 and the RTQ-10 demonstrated excellent internal consistency and good test-retest reliability (α = 0.946: r = 0.844) and (α = 0.903: r = 0.776) respectively. Also, five items from the original version were omitted due to inadequate factor loadings. This study showed that the resulting 26-item version has a two-factor structure, while the short version has a one-dimensional structure. Finally, it was found that repetitive thinking has a positive and powerful relationship with other measures of rumination and with symptoms of depression, anxiety, and stress.

**Conclusion:**

Persian versions of the RTQ have good factor structures and psychometric properties and can be used in clinical populations and related studies.

## Introduction

People with emotional disorders constantly experience repetitive thinking (RT) about past events, current problems, and future worries, which is known as repetitive negative thinking (RNT).^1^ RNT is long-lasting and recurring thoughts about one’s self, worries, and experiences that commonly occur in the minds of all individuals^2^ and are involved in development and persistence of mood and anxiety disorders.^3^ Evidence suggests that, as a subset of negative thoughts, rumination and worry have a relationship with vulnerability to various emotional disorders such as depression,^4 , 5^ post-traumatic stress disorder (PTSD),^6^ social anxiety disorder (SAD),^7 , 8^ and obsessive-compulsive disorder (OCD).^5 , 9^ RNT appears to exacerbate and prolong emotional disorders through a mechanism of maintaining attention to negative cognition and emotional contexts.^1^

Rumination is a type of RT focused on the past and has been recognized over the past two decades as an important construct in the etiology of emotional disorders, especially depression.^10^ Prospective studies have shown that depressive rumination predicts the likelihood, severity, and length of depressive symptoms.^11 , 12^ Experimental studies have also shown that depressive rumination in people who experience mood disorders causes difficulties in problem solving and negative affectivity.^13^ Evidence is increasingly pointing to the role of depressive rumination in the development of symptoms of disorders other than depression.^13 , 14^ Some evidence suggests that rumination can significantly predict anxiety and depression,^15^ bulimia nervosa,^16 , 17^ and substance abuse.^18 , 19^

Worry is another form of RT and is defined as a chain of thoughts and images containing negative emotions that are focused on the future and are relatively uncontrollable. The content of the worries generally includes recurring thoughts about possible threat and danger and catastrophic and uncertain images that are more relevant to the context of anxiety disorders, such as generalized anxiety disorder (GAD),^20^ SAD, OCD,^21^ and PTSD,^22 , 23^ in relation to which worry has been studied.^24^

Worry seems to be an attempt to avoid negative events, preparation for hazardous situations, and problem-solving, but has mostly ineffective consequences, including increased negative mood, interference with cognitive function, and impaired physiological processes.^3^

Although rumination and worry primarily differ with regard to their temporal focus (rumination is past-focused, worry is future-focused),^25^ there is some evidence to suggest that these two constructs are subsets of RT that share the characteristics of being repetitive, passive, or uncontrollable,^1 , 26^ but differ in the content of thinking: hopelessness in rumination and threat from future events in worry^1^ ; while both are reported in individuals with various clinical disorders.^27^ These similarities have also been observed in their measurement scales. Evidence has shown that symptoms of anxiety and depression can be predicted by disorder-specific questionnaires such as the Ruminative Response Scale (RRS)^28^ and the Penn State Worry Questionnaire (PSWQ).^29^ In these scales, mean scores in the range of anxiety disorders and depression were not significantly different and were highly correlated with each other.^30 – 32^

Investigating the relationship between RRS and PSWQ has shown that rumination and worry are significantly associated with anxiety and depression.^30 , 33^ Thus, RNT can be considered a transdiagnostic factor in development of emotional problems.^1^ Since RT is considered a significant risk factor for occurrence and persistence of emotional disorders, accurate measurement of this construct can be very important. Until now, different measures have been developed to assess rumination and worry. However, given the metacognitive nature of RT, none of these instruments can measure the amount of RT alone. McEvoy et al.^34^ assumed that rumination and worry overlap with RTs. The items were drawn from existing self-report questionnaires that measured components of RT (including the PSWQ-27,^29^ the RRS-28,^28^ and the PEPQ-R^35^ ). First, items that were related to depressive symptoms, physiological symptoms, and general anxiety were eliminated. Then 31 items that were related to RT were compiled and analyzed.

The resulting instrument was named the Repetitive Thinking Questionnaire (RTQ-31) and factor analysis results showed that these 31 items loaded onto two factors. The first factor, Repetitive Negative Thinking (RNT), consisted of 27 items with positive sentences and the second, Absence of Repetitive Thinking (ART) comprised the remaining 4 items.^34^ The study implicitly showed that factor analysis performed on the common items of these three questionnaires measures a general RT index.^34^

McEvoy et al.^35^ extracted a short version from the RTQ-31 for research and clinical use, the RTQ-10. The short version consisted of the 10 items with the greatest functional load. The brevity and ease of implementation of the 10-item version is a unique advantage of this questionnaire, it has shown acceptable reliability in clinical (α = 0.94) and non-clinical populations (α = 0.92), and it is highly correlated with all of the 27 items of the RNT factor.^35^ On the other hand, in the RTQ-10, respondents either respond to the questionnaires focusing on a past stressful event^34 , 36^ or on a future stressor,^37^ so RT is assessed as a time-dependent construct.^35^ Also, given the metacognitive nature of RT, implementing a short scale for this construct in clinical settings, where comorbidity of mental disorders is common, can be helpful and avoids administering different questionnaires with different guidelines.^38^

Administration of the RTQ-31 and RTQ-10 in clinical research and applications is increasing. This tool has been translated and its psychometric properties have been studied in various countries such as Japan,^39^ Turkey,^40^ and Portugal.^41^ These studies have demonstrated the psychometric adequacy of the tool. Also, in Iran, a study by Akbari^42^ examined the psychometric properties of the RTQ-10. The results showed that the short version of this questionnaire has good validity. However, the full version has not been evaluated yet in Iran. Accordingly, we aim to investigate the psychometric properties and factor structure of the Persian version of the RTQ-31 and RTQ-10 since, given the lack of sufficient evidence, the question arises of whether or not the Persian versions of the RTQ-31 and RTQ-10 have good internal structure and homogeneity in the non-clinical population, like the English versions do.^34^

## Method

### Participants

The participants in the study were 592 people (61.3% female) with a mean age of 21.5 (standard deviation [SD] = 3.38; range = 18-48) years who were undergraduate and postgraduate students selected with a convenience sampling method. Data were analyzed for statistical distribution. It was assumed that the data were normal. Being a student was the inclusion criterion and incomplete questionnaires were the exclusion criterion.

### Measures

#### Repetitive Thinking Questionnaire-31 (RTQ-31)

This questionnaire contains 31 items and was developed in 2010 by McEvoy et al. in order to measure rumination intensity. Twenty-seven items indicate RNT, and 4 items indicate an ART. Cronbach’s alpha was obtained to assess the internal consistency of the RNT (α = 0.89) and ART (α = 0.62). The instrument is scored on a 5-point Likert scale from 1 (not true at all) to 5 (very true).^34^

#### Repetitive Thinking Questionnaire-10 (RTQ-10)

The RTQ-10 is a measure consisting of 10 items selected from the main items of the RTQ-31 and scored with a 5-point Likert scale from 1 (not true at all) to 5 (very true). Evaluation of this questionnaire in both clinical and non-clinical populations showed that this measure had high internal reliability (Cronbach’s alpha greater than 0.89) and was highly correlated with the 27 items of the RNT subscale.^35^

#### Beck Depression Inventory – Second Edition

The BDI-II consists of 21 items and was developed in 1996 by Beck et al. to measure depression severity. The internal reliability of this questionnaire was high (α = 0.91) and one-week interval test-retest reliability was reported as 0.93.^43^

#### Beck Anxiety Inventory (BAI)

This 21-item questionnaire was developed in 1988 by Beck et al. to measure the severity of anxiety in adults and adolescents. They obtained a Cronbach’s alpha coefficient of 0.93 and a test-retest reliability coefficient of 0.83 for this questionnaire.^44^

#### Ruminative Response Scale (RRS)

This self-report scale, developed by Nolen-Hoeksema in 1991, measures individuals’ tendency to rumination in response to depressed mood. This scale has 22 items with four-choice Likert responses. Scale scores range from 22 to 88 and high scores indicate a high tendency for individuals to respond with rumination. Research findings have reported a test-retest coefficient of 0.67 for the ruminative response scale. The instrument also indicates individuals’ vulnerability to depression and predicts the clinical course of depression.^45^

#### Perseverative Thinking Questionnaire (PTQ)

The PTQ is a 15-item questionnaire developed by Ehring et al.^46^ that includes 3 subscales: core characteristics of RT (e.g., “The same thoughts keep going through my mind again and again”), unproductiveness (e.g., “I think about many problems without solving any of them”), and RNT capturing mental capacity (e.g., “I can’t do anything else while thinking about my problems”). This self-report questionnaire is rated on a 5-point Likert scale from 0 (never) to 4 (almost always). The full scale demonstrates excellent internal consistency (α = 0.95), while each subscale shows good internal consistency (core α = 0.94; unproductiveness α = 0.87; mental capacity α = 090).^46^

#### Depression, Anxiety, Stress Scale-21

The DASS-21 was developed by Lovibond and Lovibond in 1995 and has 21 items. Items are answered on a four-point Likert scale from zero (not at all) to 3 (very much), and a higher score indicates more severe symptoms. The depression (α = 0.94), anxiety (α = 0.87), and stress (α = 0.91) subscales all have excellent internal consistency.^47^

### Procedure

A back-translation method was used to obtain an accurate Persian version of the RTQ. Initially, the original version of this questionnaire was translated to Persian by two assistant professors of clinical psychology. In the next phase, all proposed defects were resolved. The final Persian version was then translated back into English by an English language expert and was highly consistent with the original version of the questionnaire. Finally, the Persian version of the questionnaire was reviewed by an editor to ensure that the concepts and construct of the items had been correctly translated. In the next phase, for initial evaluation, the Persian version was evaluated as a pilot with 40 students. Based on the feedback of the students who participated in the pilot evaluation, the comprehensibility of the items was re-evaluated, and a final revision was made. The final versions can be accessed in the online-only supplementary material .

### Data analyses

Assumptions of normality were checked and skew and Kurtosis were not evident in the subscales of the total scale for the normative group. Exploratory factor analysis was performed with varimax rotation to test the factor structure of the questionnaire and confirmatory factor analysis was performed to fit the model. Convergent validity was also assessed by calculating the correlation between the RTQ-10 score and the RTQ-31 total score and subscales and scores from the RRS, BDI-II, BAI, and DASS-21 questionnaires. Two methods were used to assess the stability of these two tools. Internal consistency was tested by calculating Cronbach’s alpha and test response stability was assessed by performing test-retest analysis with 32 individuals (68.8% female; age: M = 21.6 years, SD = 1.89; range = 18-28) with a 4-week interval. Statistical analyses were conducted using SPSS software version 25 and AMOS version 24.

### Ethics approval

All procedures performed in studies involving human participants were in accordance with national research committee ethical standards and with the 1964 Helsinki Declaration and its later amendments or comparable ethical standards. The study was approved by the Bioethics Committee at the Zanjan University of Medical Sciences (IR.ZUMS.REC.1398.479).

## Results

### Exploratory factor analysis of the RTQ-31

Data were analyzed by principal component analysis with varimax rotation. Initial results indicated that four factors had eigenvalues higher than 1 (11.470, 1.821, 1.189, 1.115). In order to select the most appropriate items, we excluded items with factor loadings less than 0.30. Due to this criterion, 5 items were omitted (3, 4, 6, 9, and 27) and then exploratory factor analysis was performed again ( Table 1 ). In the exploratory factor analysis of the remaining 26 items, only two factors had high eigenvalues (10.449, 1.786) and the statistical indices were appropriate for factor analysis (Kaiser-Meyer-Olkin [KMO] = 0.962; χ^2^ = 6976.992; df = 325; p < 0.005). The first factor consisted of all positive statements (n = 22), denoted as RNT, and explained 40.187% of variance. The second factor, labeled ART, includes all negative statements (n = 4) and explain 6.871% of variance.

**Table 1:** Exploratory factor analysis of the RTQ-31

Factor 1: Repetitive Negative Thinking (RNT)		Components		Mean (SD)	Cronbach’s alpha if item deleted
		RNT	ART		
1	You had thoughts or images about the situation that occurred over and over again, that resulted in your feelings getting worse and worse.	**0.639**	-0.127	2.481 (1.283)	0.923
5	You had thoughts or images about all your shortcomings, failings, faults, mistakes.	**0.634**	-0.038	2.895 (1.272)	0.923
7	Your thoughts overwhelmed you	**0.706**	-0.118	2.873 (1.375)	0.922
8	You had thoughts or images like “Why do I have problems other people don’t have?”	**0.560**	0.030	2.694 (1.451)	0.924
10	You had thoughts or images about a past event that came into your head even when you did not wish to think about it again.	**0.690**	0.017	2.944 (1.310)	0.922
11	You had thoughts or images that “I won’t be able to do my job/work because I feel so badly.”	**0.665**	-0.038	2.478 (1.282)	0.923
12	You went away by yourself and thought about why you felt this way.	**0.706**	-0.079	2.724 (1.282)	0.922
13	You had thoughts or images about the situation that resulted in you avoiding similar situations and that reinforced a decision to avoid similar situations.	**0.544**	0.238	2.665 (1.300)	0.924
15	You had thoughts or images like “Why can’t I get going?”	**0.640**	0.101	2.236 (1.290)	0.923
16	You had thoughts or images of the situation that were difficult to forget.	**0.761**	-0.021	2.807 (1.303)	0.921
17	I was always thinking about something.	**0.573**	0.064	3.070 (1.285)	0.924
19	Once I started thinking about the situation, I couldn’t stop.	**0.757**	-0.056	2.751 (1.354)	0.921
21	You had thoughts or images about how alone you felt.	**0.709**	-0.073	2.743 (1.405)	0.922
22	You had a lot of thoughts or images of the situation after it was over.	**0.751**	-0.088	2.893 (1.315)	0.921
23	I noticed that I had been thinking about the situation.	**0.723**	-0.007	3.047 (1.276)	0.922
24	You had thoughts or images of the situation that you tried to resist thinking about.	**0.722**	0.130	2.555 (1.324)	0.921
25	You had thoughts or images about how angry you were with yourself.	**0.625**	0.133	2.582 (1.286)	0.923
26	I thought about the situation all the time.	**0.680**	-0.055	2.302 (1.268)	0.926
28	I knew I shouldn’t have thought about the situation, but I couldn’t help it.	**0.773**	-0.041	2.549 (1.316)	0.921
29	You had thoughts or images asking “Why do I always react this way?”	**0.708**	0.064	2.565 (1.264)	0.922
30	You had thoughts or images about the situation and wishing it had gone better.	**0.701**	0.085	3.118 (1.342)	0.922
31	The situation really made you think	**0.758**	0.001	3.006 (1.274)	0.921
**Factor 2: Absence of Repetitive Thinking (ART)**					
2	There was nothing more I could do about the situation, so I didn’t think about it anymore.	-0.153	**0.633**	2.44 (1.23)	0.933
14	I found it easy to dismiss distressing thoughts about the situation.	-0.164	**0.629**	2.24 (1.15)	0.932
18	I didn’t tend to think about it (the situation).	0.191	**0.659**	2.57 (1.26)	0.928
20	I didn’t have enough time to do everything, so I didn’t think about it	0.085	**0.613**	1.91 (1.01)	0.929
	**Eigenvalues**	10.449	1.786		
	**% of variance**	40.187	6.871		
	**Internal consistency**	0.946	0.535		

ART = absence of repetitive thinking; RNT = repetitive negative thinking; RTQ-31 = Repetitive Thinking Questionnaire-31; SD = standard deviation.

Bold numbers loaded in each component.

### Confirmatory factor analysis of the RTQ-31

**Figure 1:**
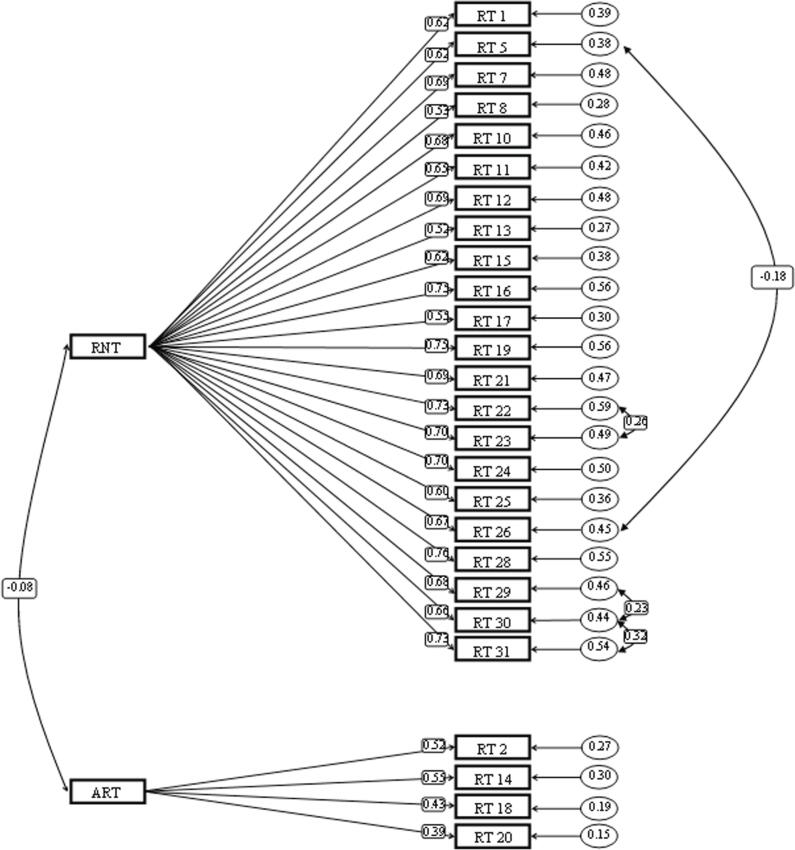
Confirmatory factor analysis of the RTQ-26. ART = absence of repetitive thinking; RNT = repetitive negative thinking; RT = repetitive thinking.

The RTQ-31 two-factor model was tested based on the work of McEvoy et al.^34^ using AMOS software, version 24. The appropriateness of the model was evaluated with the goodness-of-fit index (GFI), the Tucker-Lewis index (TLI), and the root mean square error of approximation (RMSEA). Comparative fit index (CFI) and TLI values below 0.95 and above 0.90 show acceptable goodness of fit, and RMSEA values below 0.05 indicate excellent goodness of fit.^48 , 49^ Chi-square goodness of fit indices (χ^2^ = 697.469; df = 294; p < 0.0001), CFI = 0.940, NFI = 0.902, GFI = 0. 916 and RMSEA = 0.048 show good fit. As a result, the RTQ two-factor model is well fitted, and its factor structure is confirmed ( Figure 1 ).

### Internal consistency and convergent validity of RTQ-26

Cronbach’s alpha indicates that the RNT factor has high internal consistency (α = 0.946; average inter-item correlation = 0.442), but the ART factor containing 4 items has poor internal consistency (α = 0.535; average inter-item correlation = 0.224). Internal consistency was also excellent for all 26 items (α = 0.926; average inter-item correlation = 0.317). The correlation between the RNT and ART factors is also low (Pearson’s r = -0.01). In order to assess the consistency of responses, 4 weeks after the initial test, 32 people who had answered the questionnaire in the first stage were subjected to a re-test. The results show a high re-test correlation coefficient (r = 0.844).

Pearson’s correlation coefficient revealed that the RTQ-26 has good convergent validity ( Table 2 ). The correlation coefficients for this questionnaire with the PTQ (r = 0.735) and the RRS (r = 0.663) were positive and strong, and there were positive and moderate correlations with the BDI-II (r = 0.560), the BAI (r = 0.560), and the depression (r = 0.533), anxiety (r = 0.533), and stress (r = 0.617) subscales of the DASS-21.

**Table 2:** RTQ-26 correlations, means, and standard deviations of the variables

	1	2	3	4	5	6	7	8
1. RTQ-26	-							
2. PTQ	0.735 *	-						
3. RRS	0.663 *	0.709 *	-					
4. BDI-II	0.560 *	0.590 *	0.614 *	-				
5. BAI	0.568 *	0.585 *	0.596 *	0.651 *	-			
6. DASS-D	0.533 *	0.644 *	0.711 *	0.702 *	0.599 *	-		
7. DASS-A	0.533 *	0.609 *	0.583 *	0.601 *	0.738 *	0.685 *	-	
8. DASS-S	0.668 *	0.687 *	0.724 *	0.645 *	0.635 *	0.745 *	0.729 *	-
M	69.268	22.845	45.090	13.054	13.326	5.932	4.767	7.453
SD	19.925	14.244	13.924	9.402	10.578	5.035	4.311	5.148

BAI = Beck Anxiety Inventory; BDI = Beck Depression Inventory; DASS = Depression Anxiety Stress Scale – 21 item; DASS-A = DASS – Anxiety scale; DASS-D = DASS – Depression scale; DASS-S = DASS – Stress scale; PTQ = Perseverative Thinking Questionnaire; RTQ-26 = Repetitive Thinking Questionnaire 26-Item Version.

* Correlation is significant at the p ≤ 0.001 level (2-tailed).

The *t* test was used to compare mean scores between genders. The results showed that the mean scores for the RTQ-26 (t = -3.479; Z = 0.001; N1 = 228; N2 = 364) and for the RNT subscale (t = -3.596; Z = 0.001; N1 = 228; N2 = 364) had significant differences, indicating higher mean scores in females than males, while there was no difference between men and women for the ART subscale (t = 0.611; Z = 0.001; N1 = 228; N2 = 36).

### Exploratory factor analysis of the RTQ-10

The short version of the RTQ consists of the 10 RNT items with the highest factor loading out of the total of 22 items.^34^ These 10 items were subjected to varimax rotation for principal component analysis. The results showed that only one factor had an eigenvalue higher than one (5.356) and also that the statistical indices were suitable for factor analysis (KMO = 0.942; χ^2^ = 2668.739; df = 45; p < 0.005). The findings also showed that the short one-dimensional version of the RTQ explains 53.558 percent of the total variance ( Table 3 ).

**Table 3:** Exploratory factor analysis of the RTQ-10

Items		Components	Mean (SD)	Cronbach’s alpha if item deleted
5	You had thoughts or images about all your shortcomings, failings, faults, mistakes.	**0.634**	2.895 (1.272)	0.900
10	You had thoughts or images about a past event that came into your head even when you did not wish to think about it again.	**0.719**	2.944 (1.310)	0.894
11	You had thoughts or images that “I won’t be able to do my job/work because I feel so badly.”	**0.701**	2.478 (1.282)	0.895
16	You had thoughts or images of the situation that were difficult to forget.	**0.786**	2.807 (1.303)	0.889
19	Once I started thinking about the situation, I couldn’t stop.	**0.788**	2.751 (1.354)	0.889
23	I noticed that I had been thinking about the situation.	**0.735**	3.047 (1.276)	0.893
24	You had thoughts or images of the situation that you tried to resist thinking about.	**0.730**	2.555(1.324)	0.893
26	I thought about the situation all the time.	**0.716**	2.305 (1.268)	0.895
28	I knew I shouldn’t have thought about the situation, but I couldn’t help it.	**0.798**	2.549 (1.316)	0.888
30	You had thoughts or images about the situation and wishing it had gone better.	**0.695**	3.118 (1.342)	0.896
	**Eigenvalues**	5.356		
	**% of variance**	53.558		
	**Internal consistency**	.903		

RTQ-10 = Repetitive Thinking Questionnaire 10-Item Version; SD = standard deviation.

### Confirmatory factor analysis of the RTQ-10

**Figure 2:**
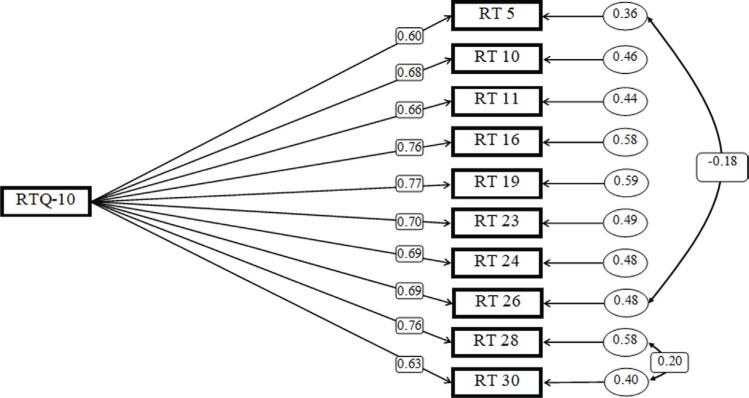
Confirmatory factor analysis of the RTQ-10. RT = repetitive thinking; RTQ-10 = Repetitive Thinking Questionnaire 10-Item Version

Confirmatory factor analysis was also tested to determine the goodness of fit of the short version one-dimensional model using AMOS software, version 24. The model fit was evaluated with GFI, TLI, and RMSEA. CFI and TLI values above 0.95 and RMSEA values below 0.05 indicate excellent goodness of fit.^48 , 49^ Chi-square fit indices (χ^2^ = 72.678; df = 33; p < 0.0001), CFI = 0.985, NFI = 0.973, GFI = 0.976 and RMSEA = 0.045 all show excellent fit. As a result, the one-factor model of the short version of the RTQ-10 has a very good fit, and its factor structure is confirmed ( Figure 2 ).

### Internal consistency and convergent validity of the RTQ-10

Internal consistency for the RTQ-10 is also excellent (α = 0.903; average inter-item correlation = 0.482). Conducting test-retest after 4 weeks also showed a correlation coefficient of r = 0.776.

Also, the correlations between this version and all the measures used in the research were significant ( Table 4 ) Separately, RTQ-10 had positive and strong correlations with the PTQ (r = 0.745), RRS (r = 0.674), BDI-II (r = 0.551), and BAI (r = 0.558). It was positively and moderately correlated with the depression (r = 0.531), anxiety (r = 0.525), and stress (r = 0.616) subscales of the DASS.

**Table 4:** RTQ-10 correlations, means, and standard deviations of the variables

	1	2	3	4	5	6	7	8
1. RTQ-10	-							
2. PTQ	0.745 *	-						
3. RRS	0.674 *	0.709 *	-					
4. BDI-II	0.551 *	0.590 *	0.614 *	-				
5. BAI	0.558 *	0.585 *	0.596 *	0.651 *	-			
6. DASS-D	0.531 *	0.644 *	0.711 *	0.702 *	0.599 *	-		
7. DASS-A	0.525 *	0.609 *	0.583 *	0.601 *	0.738 *	0.685 *	-	
8. DASS-S	0.616 *	0.687 *	0.724 *	0.645 *	0.635 *	0.754 *	0.729 *	-
Mean	27.534	22.845	45.090	13.054	13.326	5.932	4.767	7.453
SD	9.644	14.244	13.924	9.402	10.578	5.035	4.311	5.148

BAI = Beck Anxiety Inventory; BDI = Beck Depression Inventory; DASS = Depression Anxiety Stress Scale – 21 item; DASS-A = DASS – Anxiety scale; DASS-D = DASS – Depression scale; DASS-S = DASS – Stress scale; PTQ = Perseverative Thinking Questionnaire; RTQ-10 = Repetitive Thinking Questionnaire 10-Item Version; SD = standard deviation.

* Correlation is significant at the p ≤ 0.001 level (2-tailed).

The *t* test for the short version (RTQ-10) showed that there was a significant difference between the two genders (t = -3.607; Z = 0.001; N1 = 228; N2 = 364), with significantly higher mean scores for women than for men.

## Discussion

As one of the transdiagnostic factors, RT has attracted much attention in relation to occurrence and persistence of emotional disorders (anxiety disorders, depression, OCD, eating disorders). Accurate measurement of negative repetitive constructs helps to better understand the evolution of emotional disorders and their treatment. Targeting this construct as a risk factor for emotional disorders can reduce emotional maladjustment as well as reduce recurrent and other comorbidities of emotional disorders. Therefore, it is essential to have a reliable and valid measure for evaluation and diagnosis of this construct in clinical and research work. The present study aimed to investigate the psychometric properties and factor structure of the Persian versions of the RTQ-31 and RTQ-10 in the non-clinical population and to evaluate the concurrent validity of the RTQ, RRS, BDI-II, BAI, and DASS-21, adding significant evidence to previous findings.

Our findings show that both the RTQ-26 and the RTQ-10 have good psychometric properties. Confirmatory factor analysis results show that the RTQ-26 two-factor and the RTQ-10 one-dimensional model both fit well. Although the results of the initial study supported the 31-item RTQ version,^34^ five items with factor loadings of less than 0.3 were excluded in the present study and 26 items with good factor loadings remain in the Persian version.

Also, in the initial exploratory factor analysis, there were 4 eigenvalues higher than 1, but after eliminating five items with factor loadings of less than 0.3 and loading on to more than one factor, the number of eigenvalues was reduced to two factors. This strongly supports the two-factor structure of the questionnaire. The first factor is the RNT, it contains positive wording items (n = 22), and the second factor is the ART, which contains negative wording items (n = 4). The items deleted were all removed from the RNT subscale, which reduced the number of items from 27 items to 22 items. Also, by eliminating these five items, the percentage of variance explained by the RNT factor increased from 36.99% to 40.18% and the variance explained by the total questionnaire increased from 42.87% to 47.05%. Theoretically, it seems that the low factor loading of these five items is due to the cultural and linguistic incompatibility of the items with Iranian society. Consistent with previous studies, exploratory factor analysis of the RTQ-10 showed a one-dimensional structure that had excellent internal consistency and explained 53.55% of the variance.

This study showed that mean scores for RT differed between men and women. Gender differences were significant in the full version of the RTQ-26, the RNT subscale, and the short version of the RTQ-10. Mean scores for females were higher than those for males. This finding was consistent with the results of studies that examined the demographic differences of RT with diagnosis-specific measures such as the RRS^45^ and the PSWQ^50^ ; these findings implicitly indicate that the higher prevalence of emotional disorders in women could be due to their higher RT.

The convergent validity of the RTQ-26 and the RTQ-10 was confirmed by correlating their scores with those of other instruments that measure RT constructs. The results showed that both the RTQ-26 and the RTQ-10 were significantly correlated with the PTQ and the RRS. These findings are consistent with previous studies.^35 , 51^ Also, running a re-test on some of the participants showed that both questionnaires have good test-retest reliability.

Consistent with previous studies,^11 , 36 , 52^ our findings showed that RT has a positive and strong relationship with symptoms of depression, anxiety, and stress. McLaughlin and Nolen-Hoeksema^52^ showed that rumination plays the most significant role in the co-existence of mood disorders and anxiety. Also, another study^53^ showed that both the RTQ-10 and the RTQ-31 and also the RNT subscale were positively and significantly correlated with the DASS-21 depression, anxiety, and stress subscales. There is a direct and significant relationship between BDI-II and BAI.

Finally, confirmatory factor analysis showed that the Persian version of the RTQ-26 had a better fit than the 31-item version. Therefore, use of the RTQ-26 is recommended in the Iranian population. Also, confirmatory factor analysis of the Persian version of the RTQ-10 showed that it was better than the 26-item version in all fit indices. Application in psychotherapy, shortness, one-dimensional structure, and appropriate psychometric properties are among the advantages of the RTQ-10 version that may be useful in clinical practice.

As mentioned in the introduction, RNTs are an important factor in exacerbation and maintenance of multiple emotional disorders,^1 , 2^ interfering with problem-solving, and increasing negative emotion. Accordingly, in the early phase of treatment, identifying and targeting this structure improves the effectiveness of treatment and reduces the risk of recurrence and relapse. It is hoped that the present study will play an important role in developing a valid and reliable tool for identifying and evaluating this structure in clinical practice and in motivating further experimental studies in the Persian-speaking population.

### Limitations

Like other studies, our study also has limitations. It would be possible to improve the generalizability of the results by addressing these limitations in future studies. This study was conducted on a student sample, which makes it difficult to extend findings to other groups in the community, to clinical clients, or to inpatient and outpatient samples. Therefore, to overcome this limitation, the instruments could be evaluated in a study with an appropriate sample size in different clinical and non-clinical groups.

On the other hand, we had difficulty with return of questionnaires for test-retest, out of about 150 people, only 40 people returned their questionnaires and out of these 40 questionnaires, only 32 were suitable for analysis. Selecting a larger sample size as well as implementing the questionnaire in clinical settings (outside the university setting) could increase the likelihood of questionnaires being returned. This problem also made it difficult for us to properly assess divergent validity. Therefore, lack of divergent validity assessment is another limitation of the present study. In this study, we used principal component analysis for exploratory factor analysis and the maximum-likelihood method for confirmatory factor analysis. Item response theory could be used to investigate precision along the latent trait continuum.

## Supplementary material


