# Supplementary material for: Psychometric properties and factor structure of the Repetitive Thinking Questionnaire: Persian versions of the RTQ-31 and RTQ-10
Source: Trends Psychiatry Psychother. 2021 Nov 9;44:e20200058. doi: 10.47626/2237-6089-2020-0058 (PMC9907387; doi:10.47626/2237-6089-2020-0058)
Supplement: Supplementary file 1 [file 2238-0019-trends-44-e20200058-suppl01.pdf]

http://dx.doi.org/10.47626/2237-6089-2020-0058

RTQ-26 (Persian version)

موقعیت یا رویداد آشفته سازی را که اخیراً تجربه کرده‌اید، به خاطر بیاورید. به جزئیات آن فکر کنید. با توجه به تجاربی که در رابطه با این موضع داشته‌اید. میزان درستی هر یک از جملات را مشخص کنید.

	جملات	اصلاً درست نیست	کمی درست است	تا حدی درست است	اغلب درست است	کاملاً درست است
1	درباره آن موقعیت، افکار و تصاویر ذهنی داشته‌ام که بارها و بارها تکرار شده‌اند و این افکار احساساتم را بدتر و بدتر کرده‌اند.					
2	در آن شرایط کار دیگری از دستم برنمی‌آمد، بنابراین دیگر درباره آن فکر نمی‌کنم.					
3	افکار و تصاویر ذهنی درباره کم و کاستی‌ها، نقص‌ها، شکست‌ها و اشتباهاتم داشته‌ام.					
4	بعدازآن موقعیت، افکارم مرا در خود غرق کردند.					
5	افکار و تصاویر ذهنی شبیه به این داشتم که «چرا من مشکلاتی دارم که دیگران ندارند».					
6	درباره این رویداد افکار و تصاویری داشتم که وارد ذهنم می‌شدند حتی اگر نمی‌خواستم درباره آن‌ها فکر کنم.					
7	افکار و تصاویر ذهنی شبیه به این داشتم که «چون احساس خیلی بدی دارم دیگر نمی‌توانم کارم را انجام دهم».					
8	در خودم و افکارم غوطه‌ور می‌شدم تا بدانم چرا چنین احساسی دارم.					
9	درباره آن موقعیت افکار و تصاویر ذهنی داشته‌ام که باعث شدند از موقعیت‌های مشابه دوری‌کنم و تصمیم‌گیری برای اجتناب از چنین موقعیت‌هایی را بیشتر کردند.					
10	نادیده گرفتن افکار آشفته ساز درباره آن موقعیت آسان بود.					
11	افکار و تصاویر ذهنی شبیه به این داشتم: «چرا نمی‌توانم اینجا را ترک کنم».					
12	درباره آن موقعیت افکار و تصاویر ذهنی داشته‌ام که سخت می‌شد فراموششان کرد.					
13	من همیشه در حال فکر کردن به چیزهای مختلف بودم.					
14	تمایل نداشتم درباره آن موقعیت فکر کنم.					
15	وقتی فکر کردن به آن موقعیت شروع می‌شد، دیگر نمی‌توانستم افکارم را متوقف کنم.					
16	من زمان کافی برای انجام هیچ کاری را نداشتم، بنابراین درباره آن موقعیت فکر نمی‌کردم.					
17	افکار و تصاویر ذهنی داشتم دراین‌باره که چقدر احساس تنهایی می‌کنم.					
18	حتی بعد از مدت‌ها پس‌ازآن موقعیت، باز درباره آن موضوع، افکار و تصاویر ذهنی داشتم.					
19	به خاطر می‌آورم که درباره آن موقعیت زمان‌هایی را مشغول فکر کردن بودم.					
20	افکار و تصاویر ذهنی شبیه به این داشتم که: «از مقاومت در مقابل این فکرها خسته شده‌ام».					
21	افکار و تصاویر ذهنی داشتم با این محتوا که چقدر در آن موقعیت از دست خودم عصبانی بودم.					
22	در همه‌وقت و همیشه درباره آن موقعیت فکر می‌کردم.					
23	می‌دانستم که درباره آن موقعیت نباید فکری داشته باشم ولی نمی‌توانستم برای از بین بردن آن‌ها به خودم کمک کنم.					
24	افکار و تصاویر ذهنی پرسش‌کننده‌ای مثل این داشتم که «چرا من همیشه به این شیوه برخورد می‌کنم».					
25	درباره آن موقعیت افکار و تصاویر ذهنی داشتم و آرزو می‌کردم کاش اتفاق بهتری افتاده بود.					
26	آن موقعیت واقعاً من را وادار به فکر کردن می‌کند.					

http://dx.doi.org/10.47626/2237-6089-2020-0058
Psychometric properties and factor structure of the Repetitive Thinking Questionnaire: Persian versions of the RTQ-31 and RTQ-10 - Hasani et al.

RTQ-10 (Persian version)

موقعیت یا رویداد آشفته سازی را که اخیراً تجربه کرده‌اید، به خاطر بیاورید. به جزئیات آن فکر کنید. با توجه به تجاربی که در رابطه با این موضع داشته‌اید. میزان درستی هر یک از جملات را مشخص کنید.

	جملات	اصلاً درست نیست	کمی درست است	تا حدی درست است	اغلب درست است	کاملاً درست است
1	افکار و تصاویر ذهنی درباره کم و کاستی‌ها، نقص‌ها، شکست‌ها و اشتباهاتم داشته‌ام.					
2	درباره این رویداد افکار و تصاویری داشتم که وارد ذهنم می‌شدند حتی اگر نمی‌خواستم درباره آن‌ها فکر کنم.					
3	افکار و تصاویر ذهنی شبیه به این داشتم که «چون احساس خیلی بدی دارم دیگر نمی‌توانم کارم را انجام دهم».					
4	درباره آن موقعیت افکار و تصاویر ذهنی داشته‌ام که سخت می‌شد فراموششان کرد.					
5	وقتی فکر کردن به آن موقعیت شروع می‌شد، دیگر نمی‌توانستم افکارم را متوقف کنم.					
6	به خاطر می‌آورم که درباره آن موقعیت زمان‌هایی را مشغول فکر کردن بودم.					
7	افکار و تصاویر ذهنی شبیه به این داشتم که: «از مقاومت در مقابل این فکرها خسته شده‌ام».					
8	در همه‌وقت و همیشه درباره آن موقعیت فکر می‌کردم.					
9	می‌دانستم که درباره آن موقعیت نباید فکری داشته باشم ولی نمی‌توانستم برای از بین بردن آن‌ها به خودم کمک کنم.					
10	درباره آن موقعیت افکار و تصاویر ذهنی داشتم و آرزو می‌کردم کاش اتفاق بهتری افتاده بود.					
